# Association Study of IL-12B Polymorphisms Susceptibility with Ankylosing Spondylitis in Mainland Han Population

**DOI:** 10.1371/journal.pone.0130982

**Published:** 2015-06-23

**Authors:** Li Zhang, Dazhi Fan, Li Liu, Ting Yang, Ning Ding, Yanting Hu, Guoqi Cai, Li Wang, Lihong Xin, Qing Xia, Xiaona Li, Shengqian Xu, Jianhua Xu, Xiao Yang, Yanfeng Zou, Faming Pan

**Affiliations:** 1 Medical Genetics Center in Anhui Medical College, Hefei, 230061, China; 2 Department of Epidemiology and Biostatistics, School of Public Health, Anhui Medical University, 81 Meishan Road, Hefei, Anhui, 230032, China; 3 Department of Rheumatism and Immunity, the First Affiliated Hospital of Anhui Medical University, Hefei, Anhui, 230022, China; Taipei Medicine University, TAIWAN

## Abstract

**Objective:**

This study aims to determine whether the genetic polymorphisms of IL-12B gene is a susceptibility factor to Ankylosing spondylitis (AS) in mainland Han Chinese population.

**Method:**

Eight single-nucleotide polymorphisms (SNPs) (rs10045431, rs11167764, rs3212227, rs6556412, rs6556416, rs6871626, rs6887695 and rs7709212) in the IL-12B gene were genotyped by iMLDR Assay technology in 400 patients [96% (384/400) HLA-B27(+)] and 395 geographically and ethnically matched healthy controls in mainland Han Chinese population. The correlation between IL-12B genetic polymorphisms and AS activity index (BASDAI, BASFI) were tested.

**Results:**

The significant difference was found in genotype distribution between AS and healthy controls (χ^2^ = 6.942, *P*-value = 0.031) of the SNP rs6871626. Furthermore, significant evidence was also detected under the recessive model for minor allele A. The AA genotype carrier had 1.830 fold risk compared with C allele carrier (with CC and AC genotypes) [OR (95% CI) = 1.830 (1.131-2.961), *P*-value = 0.014]. Nevertheless, the difference was no longer significant after Bonferroni correction. Subset analysis on cases with HLA-B27(+) did find the same results. Three genotypic groups (AA, CC and CA) in rs6871626 site was highly associated with the BASDAI and BASFI (*P*-value = 0.012 and *P*-value = 0.023, respectively), after adjustment for effect of age, sex, and disease duration, the *P*-value was 0.031 and 0.041, respectively. The AA genotype of rs6871626 was also significantly correlated with an increased BASDAI and BASFI compared to the AC and CC genotypes in AS patients.

**Conclusion:**

Our findings suggest that rs6871626 may be associated AS susceptibility and with disease activity (BASDAI, BASFI) in mainland Han Chinese population.

## Introduction

Ankylosing Spondylitis (AS) is a chronic inflammatory arthritis that mainly affects the sacroiliac joints and spinal joints, causing pain and stiffness, and progressive fusion of involved joints, it is characterized by axial skeletal inflammation, enthesis and iritis, and mainly affects young men with higher clinical incidence and family aggregation [1, 2]. So far, the precise pathogenesis of AS is unclear, but some studies [3, 4] suggested that HLA-B27 gene was associated with the susceptibility of AS. In addition to the HLA region, the Genome-Wide Association Studies (GWAS)[5, 6] and our group’s work also suggested the AS correlation of non-HLA regions, such as IL-33 [7], FCGR2B [8], FCRL4b [9], KIR3DS1 [10].

IL-12B gene is located in non-HLA region of chromosome 5q31–33, length of 15kb, containing eight exons, and its expression levels are mainly post-transcriptionally regulated [11]. IL-12 is an N-acylated sugars heterodimer, constituting the p35 (coded for by IL-12A) and p40 (coded for by IL-12B) subunits. It is of crucial relevance to cell-mediated immunity and Th1 differentiation [12], and plays a central role in promoting the differentiation of naive CD4^+^ T cells into mature interferon-γ producing T-helper (Th1) effect cells. Moreover, it is a potent stimulus of natural killer (NK) and CD8^+^ T cells for IFN-γ production [13]. IL-12B, a subunit of IL-12, can induce expression of interferon-γ, leading to the differentiation and proliferation of Th1 [14]. IL-12B knockout mice, which were defective in both the IL-12 and IL-23 pathways, were not susceptible to experimentally induced autoimmune encephalomyelitis [15]. This suggests that it may play a key role in this pathway. The gene single-nucleotide polymorphisms (SNPs) may affect the function and expression of IL-12B. Many disease-related studies on IL-12 gene polymorphisms were more focused on the study of IL-12B gene polymorphism.

IL-12B gene has been implicated in the pathogenesis of a multitude of diverse autoimmune diseases [16–19], such as Psoriasis, Systemic lupus erythematosus (SLE), Crohn’s disease (CD) and Ulcerative colitis (UC). Previously several SNPs in IL-12B gene have been tested in AS, however, the results were controversial [5, 20–24]. It is unclear whether some of SNPs in IL-12B gene are associated with AS susceptibility and others are associated with disease severity. In this study, we selected eight SNPs (four of eight have been reported) to test whether candidate genetic variations contribute to AS susceptibility and severity in mainland Han Chinese population (Anhui province).

## Materials and Methods

### Subjects

A total of 400 patients with AS [96% (384/400) HLA-B27(+)], fulfilling the modified New York Criteria (1984)[25], and 395 geographically and ethnically matched healthy controls were enrolled in the current study. All healthy controls were interviewed to exclude any history of AS disorder. The disease duration was defined as the time from the first symptom (inflammatory back pain, peripheral arthritis, uveitis, or enthesitis) was noted to the time when enrolled in the current study. All subjects are from mainland Han Chinese population (Anhui province). This study conformed to the Declaration of Helsinki, and the design of the work was reviewed and approved by the ethics committee of Anhui Medical University. All the participants provided their written informed consent to join in this study.

### Genotyping

In this study, we selected SNPs of IL-12B with minor allele frequency > 5% from the Han Chinese in Beijing (CHB) population in the HapMap database (http://www.hapmap.org). Meanwhile, SNPs from previous GWASs which were associated with AS but did not test in mainland Han Chinese population were also considered. Eventually, we included eight SNPs (rs10045431, rs11167764, rs3212227, rs6556412, rs6556416, rs6871626, rs6887695 and rs7709212). One polymorphism (rs3212227) located in the 3’-UTR-exon8, one polymorphism (rs11167764) is in the 3’-flank, and other six polymorphisms are in the 5’-flank. A graphical overview of genotyped polymorphisms was shown in Figure A in S1 File. Genomic DNA was extracted from peripheral blood lymphocytes by using a commercially available kit (QIAGEN, Hilden, Germany). DNA samples were stored at -20°C before genotyping. The genotyping of SNPs were carried out by Shanghai Genesky Bio-Tech Co., Ltd. (http://biotech.geneskies.com/index.html) using the improved multiplex ligase detection reaction (iMLDR) method, the primers are listed in Table 1.

**Table 1:** SNPs and PCR primer for IL-12B allele genotyping.

SNPs	Chromosome position	PCR primer
rs10045431	158814533	rs10045431F:TGTCACCTTCAACTTGGCCTGA
		rs10045431R:ACTCCCACGTACCCCATGAGAA
rs11167764	141479065	rs11167764F:TGTTAGTGACAACCCTTGGAAGGAA
		rs11167764R:TTTATGCACTTGATCCTGAAAGGATTT
rs3212227	158742950	rs3212227F:GGCAACTTGAGAGCTGGAAAATCT
		rs3212227R:CCCASATCAACTTTTGGCATTCTC
rs6556412	158787385	rs6556412F:AATCGTTTGAGCCCAGGAGATG
		rs6556412R:TCCCACTCTTCCCTCTGAGTCC
rs6556416	158818745	rs6556416F:TCAACTGCATGGTGGGGTCAAC
		rs6556416R:AGCTGAGGCCACCCAAACTAAA
rs6871626	158826792	rs6871626F:GAGGCCAATAATCRGGCTGAAG
		rs6871626R:AGAGAGGTGAGCCGAGGCAGAG
rs6887695	158822645	rs6887695F:GGGCTTCAGGCTTCACCAGTCT
		rs6887695R:CACCCCTGAAGCGAGGWCAAAT
rs7709212	158764177	rs7709212F:TTCTCCTGGGATGGATGCATTT
		rs7709212R:TCTGCCTCCAGGAAATACCACAC

SNPs: Single nucleotide polymorphisms; PCR: polymerase chain reaction; F: forward; R: reverse.

### Assessment criteria

We assessed the following data for all patients: Disease activity was measured by the Bath Ankylosing Spondylitis Disease Activity Index (BASDAI) from 0 to 10: where 0 = no activity and 10 = maximum activity. Functional impairment was measured by the Bath Ankylosing Spondylitis Functional Index (BASFI) from 0 to 10. Higher values of BASFI indicate worse functional ability. The modified Chinese versions of BASDAI and BASFI have a good intraclass correlations and Cronbach's alpha measure [26].

### Statistical analysis

The differences in allele and genotype frequencies between cases and controls were assessed by χ^2^ tests (Fisher’s exact test was applied if the expected frequency was less than 5). Odds ratios (ORs) were calculated with 95% confidence intervals (95% CIs). Continuous data were given as mean ± standard deviation (SD). Comparisons among the three genotypic groups were calculated using One-Way ANOVA analysis [Clinical phenotype (BASFI or BASDAI) as the dependent variable and the three genotypic groups in each site as independent variables] and multiple regression analysis was used to adjust for age, sex and disease duration [Clinical phenotype (BASFI or BASDAI) as the dependent variable, the three genotypic groups in each site as independent variables and age, sex, disease duration as covariates]. The above analyses were conducted using SPSS version 10.01. Bonferroni adjustment for multiple testing was applied and *P*-value for a truly significant result was calculated as 0.05/n. Hardy-Weinberg equilibrium (HWE) was evaluated in control groups by χ^2^ test.

## Results

Four hundred patients (258 males and 142 females, mean ± SD age 28.9 ± 8.9 years), and 395 controls (255 males and 140 females, mean ± SD age 27.9 ± 7.9 years) were enrolled in our data. In cases group, the mean disease duration was 7.68 ± 1.59 years. And the mean BASDAI and BASFI scores were 4.01 ± 1.95 and 2.87 ± 2.07, respectively. There were no statistically significant differences between the groups with respect to age (t = 1.560, *P*-value = 0.119) and sex (χ^2^ = 0.001, *P*-value = 0.987).

All of the SNPs were in HWE in controls. The allele and genotype frequencies of IL-12B gene polymorphisms in cases and healthy controls were shown in Table 2. At rs6871626 locus, the significant difference was found in genotype distribution between AS and healthy controls (χ^2^ = 6.942, *P*-value = 0.031). Furthermore, significant evidence was also detected under the recessive model for minor allele A, the AA genotype carrier had 1.830 fold risk compared with C allele carrier (contained CC and AC genotypes) [OR (95% CI) = 1.830 (1.131–2.961), *P*-value = 0.014]. However, there was no significant difference after Bonferroni correction. Other SNPs were all not associated with AS susceptibility. In addition, subset analysis on cases with HLA-B27(+) did find the same results (Table A in S1 File).

**Table 2:** The allele and genotype frequencies of IL-12B gene polymorphisms in AS cases and controls.

SNPs		HWE		Allele		Genotypic frequency					Allele frequency				
	Group	χ^2^	*p* value	1	2	11	12	22	χ^2^	*p* value	1	2	χ^2^	*p* value	OR (95%CI)
rs10045431	Case			A	C	9(0.022)	75(0.188)	316(0.790)	3.411	0.182	93(0.116)	707(0.884)	2.156	0.142	1.273(0.922–1.757)
	Control	0.076	0.783			3(0.008)	68(0.172)	324(0.820)			74(0.094)	716(0.906)			
rs11167764	Case			A	C	4(0.010)	95(0.238)	301(0.752)	2.512	0.285	103(0.129)	697(0.871)	1.585	0.208	0.833(0.627–1.107)
	Control	0.002	0.988			9(0.023)	101(0.256)	285(0.722)			119(0.151)	671(0.849)			
rs3212227	Case			G	T	73(0.182)	196(0.490)	131(0.328)	0.535	0.765	342(0.427)	458(0.573)	0.223	0.637	0.953(0.7821–1.163)
	Control	0.599	0.438			80(0.203)	187(0.473)	128(0.324)			347(0.439)	443(0.561)			
rs6556412	Case			A	G	68(0.170)	201(0.503)	131(0.328)	0.209	0.901	337(0.421)	463(0.579)	0.101	0.751	0.968(0.793–1.182)
	Control	0.010	0.919			72(0.182)	195(0.494)	128(0.324)			339(0.421)	451(0.571)			
rs6556416	Case			A	C	7(0.018)	75(0.188)	318(0.795)	1.967	0.374	89(0.111)	711(0.889)	1.335	0.247	1.211(0.875–1.677)
	Control	0.076	0.783			3(0.008)	68(0.172)	324(0.820)			74(0.094)	716(0.906)			
rs6871626	Case			A	C	49(0.122)	156(0.390)	195(0.487)	6.942	0.031	254(0.318)	546(0.682)	1.063	0.302	1.119(0.904–1.385)
	Control	2.164	0.141			28(0.071)	176(0.446)	191(0.484)			232(0.294)	558(0.706)			
rs6887695	Case			C	G	61(0.152)	200(0.500)	139(0.347)	0.338	0.844	322(0.403)	478(0.598)	0.265	0.607	0.949(0.777–1.159)
	Control	0.188	0.665			66(0.167)	196(0.496)	133(0.337)			328(0.415)	462(0.585)			
rs7709212	Case			C	T	77(0.193)	195(0.487)	128(0.320)	1.332	0.514	349(0.436)	451(0.564)	1.067	0.302	0.901(0.739–1.098)
	Control	0.898	0.343			89(0.225)	187(0.473)	119(0.301)			365(0.462)	425(0.538)			

SNPs: Single nucleotide polymorphisms; HWE: Hardy-Weinberg equilibrium; OR:odds ratio.

*P*-value was survived after Bonferroni correction.

We investigated the relationship between genetic polymorphisms and clinical phenotypes including the BASDAI and BASFI. As shown in Table 3, we found that rs6871626 was highly associated with the BASDAI and BASFI (*P*-value = 0.012 and *P*-value = 0.023, respectively), after adjustment for effect of age, sex, and disease duration, the *P*-value was 0.031 and 0.041, respectively. We further found that the AA genotype was significantly correlated with an increased BASDAI and BASFI compared to the AC and CC genotypes in AS patients (*P*-value = 0.045 and 0.009, respectively). The mean ± SD of the BASDAI for the AA genotype and combined AC and CC genotypes were 4.244 ± 1.713 and 3.655 ± 1.952, respectively. And, the BASFI were 3.058 ± 2.276 and 2.244 ± 2.013, respectively. In addition, the results did not change in HLA-B27(+) AS patients (Figure B–E in S1 File)

**Table 3:** BASDAI and BASFI scores in patients with IL-12B genotypes.

SNPs	Genetype	BASDAI	BASFI
rs10045431	A/A	4.498 ± 1.905	3.826 ± 3.201
	C/C	3.962 ± 1.912	2.891 ± 2.106
	C/A	4.154 ± 2.097	2.675 ± 1.707
	*p* value	0.557	0.271
	*p* value*	0.614	0.354
rs11167764	A/A	4.305 ± 1.079	4.545 ± 3.507
	C/C	4.087 ± 1.995	2.897 ± 2.097
	C/A	3.754 ± 1.812	2.716 ± 1.904
	*p* value	0.335	0.203
	*p* value*	0.397	0.312
rs3212227	A/A	4.011 ± 1.942	3.023 ± 2.341
	C/C	4.144 ± 2.218	2.882 ± 2.262
	C/A	3.959 ± 1.844	2.766 ± 1.782
	*p* value	0.786	0.548
	*p* value*	0.869	0.611
rs6556412	A/A	3.857 ± 1.968	2.957 ± 2.169
	C/C	4.035 ± 1.912	2.756 ± 2.069
	C/A	4.043 ± 1.971	2.902 ± 2.030
	*p* value	0.780	0.755
	*p* value*	0.852	0.877
rs6556416	A/A	3.963 ± 1.474	3.656 ± 3.565
	C/C	3.977 ± 1.921	2.901 ± 2.106
	C/A	4.154 ± 2.097	2.675 ± 1.707
	*p* value	0.775	0.419
	*p* value*	0.831	0.522
rs6871626	A/A	4.244 ± 1.713	3.058 ± 2.013
	C/C	3.983 ± 1.931	2.918 ± 2.163
	C/A	3.373 ± 1.694	2.129 ± 1.352
	*p* value	0.012	0.023
	*p* value*	0.031	0.041
rs6887695	G/G	3.873 ± 1.929	2.641 ± 1.896
	C/C	4.021 ± 1.945	2.984 ± 2.108
	G/C	4.054 ± 1.954	2.812 ± 2.085
	*p* value	0.829	0.482
	*p* value*	0.935	0.549
rs7709212	C/C	4.024 ± 1.882	2.774 ± 2.089
	C/T	4.009 ± 2.140	3.067 ± 2.291
	T/T	4.001 ± 1.916	2.858 ± 1.964
	*p* value	0.995	0.612
	*p* value*	0.999	0.786

BASDAI: Bath Ankylosing Spondylitis Disease Activity Index; BASFI: Bath Ankylosing Spondylitis Functional Index.

* Adjusting the effects of age, sex, disease duration.

## Discussion

Although the precise etiology of AS remains to be elucidated, it is widely accepted that the disease is caused by multiple factors. IL-12B gene, in addition to HLA-B27, IL-23R, ERAP1, and 2p15 region [5, 23], was postulated for the pathogenesis of AS. Our findings further indicate that the gene of IL-12B is associated with AS, and that rs6871626 may be a risk SNP involved in AS genetic predisposition and disease severity in mainland Han population (Anhui province).

SNP rs6871626 is located within a small LD block of 40kb on 5q33 and in the 5’-flank of IL-12B gene [27]. It was also reported to be associated with ulcerative colitis [28]. Genetic variations may interfere with mRNA stability or protein translation by interacting with microRNAs [29]. It has been found that aberrant expression of microRNAs contributed to the immunopathogenesis of patients with ankylosing spondylitis [30]. The association between rs6871626 and clinical manifestations of AS suggests that the fundamental may effect of IL-12B protein on AS progression as well as AS onset and the association may be more evident in a recessive manner. This raises a possibility that those with two risk alleles (AA) in rs6871626 have stronger disease serverity than the cases with single risk alleles. Understanding the functional role of rs6871626 polymorphisms in the regulation of AS susceptibility can lead to further insight into the disease pathogenesis. The mechanism needs to be further investigated.

The SNPs and AS susceptibility and severity have been reported in different ethnicity. Many studies were carried out in Taiwanese population with similar genetic background to mainland Han Chinese population. Wen et al.[31] confirmed that in Taiwanese population, the rs10865331 was associated with AS susceptibility and disease activity (BASFI). Other significant loci, including ORAI1 (rs12313273 and rs7135617)[32] and STIM1 (rs3750996)[33], were also found association with the pathogenesis of AS. In this study, we examined the correlation between the IL-12B gene SNPs and susceptibility to AS in mainland Han Chinese population. IL-12B has been demonstrated that differences in genotype and allele frequencies of cytokine gene polymorphisms depend on ethnicity and race [34]. Many studies have evaluated the association of IL-12B polymorphisms with AS risk in part of the population. Of the polymorphisms studied, the rs3212227 CC/AC genotype was more frequent among the Taiwanese patients [20], however, by detecting the complementary chain, there was no significant differences of genotypic or allelic frequencies in mainland Han Chinese in this study. The genotype frequencies of the SNP were seemingly similar in the two studies with a relatively larger sample size. Whilst one can assume that Mainland Chinese and Taiwanese populations have similar genetic backgroud the difference in this case was observed, presumably due to genetic drift. Similar results were also reported in AS association studies [23, 35–37]. Evans [5] found a positive association between the rs6556416 polymorphism with AS in European populations. However, this was of non-significant difference, probably due to a limited sample size. In terms of the observed genotype frequencies, a total of 795 patients and controls should be necessary to reach a power of 80% at a *P*-value = 0.05. We reason that this discrepancy could be attributed to ethnic difference.

We are aware that the SNPs selected in this study may be not adequate to investigate the entire genetic polymorphisms of IL-12B, and the sample size in the study may be under-powered to detect the small genetic effect of IL-12B in the disease activity such as BASDAI/BASFI, since after the Bonferroni correction, the difference turns out to be insignificant. The study needs to be replicated in another population with a larger sample size or with meta-analysis. In conclusion, our research suggests that rs6871626 in the IL-12B gene may be associated with AS susceptibility and disease activity (BASDAI and BASFI) in mainland Han Chinese population (Anhui province).

## Supporting Information

S1 File The allele and genotype frequencies of IL-12B gene polymorphisms in HLA-B27(+) AS cases and controls (Table A). The IL-12B gene structure of the selected SNPs (Figure A). Comparison of the BASDAI scores among different genotypes of rs6871626 in AS patients (Figure B). Comparison of the BASFI scores among different genotypes of rs6871626 in AS patients (Figure C). Comparison of the BASDAI scores among different genotypes of rs6871626 in HLA-B27(+) AS patients (Figure D). Comparison of the BASFI scores among different genotypes of rs6871626 in HLA-B27(+) AS patients (Figure E).
(DOC)

S1 Supporting Data The data of the genotype frequencies of IL-12B gene polymorphisms in AS cases and controls. (XLS)

## References

[1] Brown MA, Laval SH, Brophy SCalin A. Recurrence risk modelling of the genetic susceptibility to ankylosing spondylitis. Ann Rheum Dis. 2000;59:883–6. 11053066 10.1136/ard.59.11.883 PMC1753017

[2] Haywood KL, Garratt AM, Jordan K, Dziedzic KDawes PT. Spinal mobility in ankylosing spondylitis: reliability, validity and responsiveness. Rheumatology (Oxford). 2004;43:750–7. 15163832 10.1093/rheumatology/keh169

[3] Yang T, Duan Z, Wu S, Liu S, Zeng Z, Li G, et al Association of HLA-B27 genetic polymorphisms with ankylosing spondylitis susceptibility worldwide: a meta-analysis. Mod Rheumatol. 2014;24:150–61. 10.3109/14397595.2013.852856 24261772

[4] Vegvari A, Szabo Z, Szanto S, Glant TT, Mikecz K, Szekanecz Z. The genetic background of ankylosing spondylitis. Joint Bone Spine. 2009;76:623–8. 10.1016/j.jbspin.2009.02.006 19541528

[5] Evans DM, Spencer CC, Pointon JJ, Su Z, Harvey D, Kochan G, et al Interaction between ERAP1 and HLA-B27 in ankylosing spondylitis implicates peptide handling in the mechanism for HLA-B27 in disease susceptibility. Nat Genet. 2011;43:761–7. 10.1038/ng.873 21743469 PMC3640413

[6] Australo-Anglo-American Spondyloarthritis C, Reveille JD, Sims AM, Danoy P, Evans DM, Leo P, et al Genome-wide association study of ankylosing spondylitis identifies non-MHC susceptibility loci. Nat Genet. 2010;42:123–7. 10.1038/ng.513 20062062 PMC3224997

[7] Fan D, Ding N, Yang T, Wu S, Liu S, Liu L, et al Single nucleotide polymorphisms of the interleukin-33 (IL-33) gene are associated with ankylosing spondylitis in Chinese individuals: a case-control pilot study. Scand J Rheumatol. 2014;1–22. 10.3109/03009742.2013.805242 24825247

[8] Duan ZH, Pan FM, Zeng Z, Zhang TC, Wang S, Li GX, et al The FCGR2B rs10917661 polymorphism may confer susceptibility to ankylosing spondylitis in Han Chinese: a case-control study. Scand J Rheumatol. 2012;41:219–22. 10.3109/03009742.2011.625972 22416796

[9] Zeng Z, Duan Z, Zhang T, Wang S, Li G, Mei Y, et al Association of FCRL4 polymorphisms on disease susceptibility and severity of ankylosing spondylitis in Chinese Han population. Clin Rheumatol. 2012;31:1449–54. 10.1007/s10067-012-2028-y 22777505

[10] Fan D, Liu S, Yang T, Wu S, Wang S, Li G, et al Association between KIR polymorphisms and ankylosing spondylitis in populations: A meta-analysis. Mod Rheumatol. 2014; 10.3109/14397595.2014.894489 24673577

[11] Warrington JA, Bengtsson U. High-resolution physical mapping of human 5q31-q33 using three methods: radiation hybrid mapping, interphase fluorescence in situ hybridization, and pulsed-field gel electrophoresis. Genomics. 1994;24:395–8. 7698768 10.1006/geno.1994.1636

[12] Collison LW, Vignali DA. Interleukin-35: odd one out or part of the family? Immunol Rev. 2008;226:248–62. 10.1111/j.1600-065X.2008.00704.x 19161429 PMC2631363

[13] Di Cesare A, Di Meglio P, Nestle FO. The IL-23/Th17 axis in the immunopathogenesis of psoriasis. J Invest Dermatol. 2009;129:1339–50. 10.1038/jid.2009.59 19322214

[14] Trinchieri G, Pflanz S, Kastelein RA. The IL-12 family of heterodimeric cytokines: new players in the regulation of T cell responses. Immunity. 2003;19:641–4. 14614851 10.1016/s1074-7613(03)00296-6

[15] Becher B, Durell BG, Noelle RJ. Experimental autoimmune encephalitis and inflammation in the absence of interleukin-12. Journal of Clinical Investigation. 2002;110:493–497. 12189243 10.1172/JCI15751 PMC150420

[16] Kastelein RA, Hunter CA, Cua DJ. Discovery and biology of IL-23 and IL-27: related but functionally distinct regulators of inflammation. Annu Rev Immunol. 2007;25:221–42. 17291186 10.1146/annurev.immunol.22.012703.104758

[17] Paunovic V, Carroll HP, Vandenbroeck K, Gadina M. Signalling, inflammation and arthritis: crossed signals: the role of interleukin (IL)-12, -17, -23 and -27 in autoimmunity. Rheumatology (Oxford). 2008;47:771–6. 10.1093/rheumatology/kem352 18238793

[18] Fuss IJ, Becker C, Yang Z, Groden C, Hornung RL, Heller F, et al Both IL-12p70 and IL-23 are synthesized during active Crohn's disease and are down-regulated by treatment with anti-IL-12 p40 monoclonal antibody. Inflamm Bowel Dis. 2006;12:9–15. 16374252 10.1097/01.mib.0000194183.92671.b6

[19] International Multiple Sclerosis Genetics C, Wellcome Trust Case Control C, Sawcer S, Hellenthal G, Pirinen M, Spencer CC, et al Genetic risk and a primary role for cell-mediated immune mechanisms in multiple sclerosis. Nature. 2011;476:214–9. 10.1038/nature10251 21833088 PMC3182531

[20] Wong RH, Wei JC, Huang CH, Lee HS, Chiou SY, Lin SH, et al Association of IL-12B genetic polymorphism with the susceptibility and disease severity of ankylosing spondylitis. J Rheumatol. 2012;39:135–40. 10.3899/jrheum.110613 22045842

[21] Zhou L, Zhang JY, Liu Y, Zhu XQ, Zhang YH, Yang ZH, et al Novel association between IL-12B gene polymorphism and risk of ankylosing spondylitis in Ningxia population. Int J Lab Med. 2013;34:1372–1375.

[22] Danoy P, Pryce K, Hadler J, Bradbury LA, Farrar C, Pointon J, et al Association of variants at 1q32 and STAT3 with ankylosing spondylitis suggests genetic overlap with Crohn's disease. PLoS Genet. 2010;6:e1001195 10.1371/journal.pgen.1001195 21152001 PMC2996314

[23] Lin Z, Bei JX, Shen M, Li Q, Liao Z, Zhang Y, et al A genome-wide association study in Han Chinese identifies new susceptibility loci for ankylosing spondylitis. Nat Genet. 2012;44:73–7. 10.1038/ng.1005 22138694

[24] International Genetics of Ankylosing Spondylitis C, Cortes A, Hadler J, Pointon JP, Robinson PC, Karaderi T, et al Identification of multiple risk variants for ankylosing spondylitis through high-density genotyping of immune-related loci. Nat Genet. 2013;45:730–8. 10.1038/ng.2667 23749187 PMC3757343

[25] van der Linden S, Valkenburg HA, Cats A. Evaluation of diagnostic criteria for ankylosing spondylitis. A proposal for modification of the New York criteria. Arthritis Rheum. 1984;27:361–8. 6231933 10.1002/art.1780270401

[26] Wei JC, Wong RH, Huang JH, Yu CT, Chou CT, Jan MS, et al Evaluation of internal consistency and re-test reliability of Bath ankylosing spondylitis indices in a large cohort of adult and juvenile spondylitis patients in Taiwan. Clin Rheumatol. 2007;26:1685–91. 17286214 10.1007/s10067-007-0573-6

[27] Liu H, Irwanto A, Tian H, Fu X, Yu Y, Yu G, et al Identification of IL18RAP/IL18R1 and IL12B as leprosy risk genes demonstrates shared pathogenesis between inflammation and infectious diseases. Am J Hum Genet. 2012;91:935–41. 10.1016/j.ajhg.2012.09.010 23103228 PMC3487119

[28] Anderson CA, Boucher G, Lees CW, Franke A, D'Amato M, Taylor KD, et al Meta-analysis identifies 29 additional ulcerative colitis risk loci, increasing the number of confirmed associations to 47. Nat Genet. 2011;43:246–52. 10.1038/ng.764 21297633 PMC3084597

[29] Nilsen TW. Mechanisms of microRNA-mediated gene regulation in animal cells. Trends Genet. 2007;23:243–9. 17368621 10.1016/j.tig.2007.02.011

[30] Lai NS, Yu HC, Chen HC, Yu CL, Huang HB, Lu MC. Aberrant expression of microRNAs in T cells from patients with ankylosing spondylitis contributes to the immunopathogenesis. Clin Exp Immunol. 2013;173:47–57. 10.1111/cei.12089 23607629 PMC3694534

[31] Wen YF, Wei JC, Hsu YW, Chiou HY, Wong HS, Wong RH, et al rs10865331 associated with susceptibility and disease severity of ankylosing spondylitis in a Taiwanese population. PLoS One. 2014;9:e104525 10.1371/journal.pone.0104525 25184745 PMC4153545

[32] Wei JC, Yen JH, Juo SH, Chen WC, Wang YS, Chiu YC, et al Association of ORAI1 haplotypes with the risk of HLA-B27 positive ankylosing spondylitis. PLoS One. 2011;6:e20426 10.1371/journal.pone.0020426 21674042 PMC3106015

[33] Wei JC, Hung KS, Hsu YW, Wong RH, Huang CH, Jan MS, et al Genetic polymorphisms of stromal interaction molecule 1 associated with the erythrocyte sedimentation rate and C-reactive protein in HLA-B27 positive ankylosing spondylitis patients. PLoS One. 2012;7:e49698 10.1371/journal.pone.0049698 23272049 PMC3522685

[34] Chen H, Cheng S, Wang J, Cao C, Bunjhoo H, Xiong W, et al Interleukin-12B rs3212227 polymorphism and cancer risk: a meta-analysis. Mol Biol Rep. 2012;39:10235–42. 10.1007/s11033-012-1899-y 23065198

[35] Wei JC, Hsu YW, Hung KS, Wong RH, Huang CH, Liu YT, et al Association study of polymorphisms rs4552569 and rs17095830 and the risk of ankylosing spondylitis in a Taiwanese population. PLoS One. 2013;8:e52801 10.1371/journal.pone.0052801 23308121 PMC3537770

[36] Wang CM, Ho HH, Chang SW, Wu YJ, Lin JC, Chang PY, et al ERAP1 genetic variations associated with HLA-B27 interaction and disease severity of syndesmophytes formation in Taiwanese ankylosing spondylitis. Arthritis Res Ther. 2012;14:R125 10.1186/ar3855 22632381 PMC3446506

[37] Davidson SI, Liu Y, Danoy PA, Wu X, Thomas GP, Jiang L, et al Association of STAT3 and TNFRSF1A with ankylosing spondylitis in Han Chinese. Ann Rheum Dis. 2011;70:289–92. 10.1136/ard.2010.133322 21068102

