# Supplementary material for: Association Study of IL-12B Polymorphisms Susceptibility with Ankylosing Spondylitis in Mainland Han Population
Source: PLoS One. 2015 Jun 23;10(6):e0130982. doi: 10.1371/journal.pone.0130982 (PMC4477880; doi:10.1371/journal.pone.0130982)
Supplement: S1 File — The IL-12B gene structure of the selected SNPs (Figure A). Comparison of the BASDAI scores among different genotypes of rs6871626 in AS patients (Figure B). Comparison of the BASFI scores among different genotypes of rs6871626 in AS patients (Figure C). Comparison of the BASDAI scores among different genotypes of rs6871626 in HLA-B27(+) AS patients (Figure D). Comparison of the BASFI scores among different genotypes of rs6871626 in HLA-B27(+) AS patients (Figure E). (DOC) [file pone.0130982.s001.doc]

**Supplementary section**

**Table A. The allele and genotype frequencies of IL-12B gene polymorphisms in HLA-B27(+) AS case**s and controls.

| SNPs |  |  | HWE | |  | Allele | |  | Genotypic frequency | | | | |  | Allele frequency | | | | |
| --- | --- | --- | --- | --- | --- | --- | --- | --- | --- | --- | --- | --- | --- | --- | --- | --- | --- | --- | --- |
|  |  | Group | χ^2^ | *p* value |  | 1 | 2 |  | 11 | 12 | 22 | χ^2^ | *p* value |  | 1 | 2 | χ^2^ | *p* value | OR (95%CI) |
| rs10045431 |  | Case |  |  |  | A | C |  | 9(0.023) | 73(0.190) | 302(0.786) | 3.796 | 0.150 |  | 91(0.119) | 677(0.882) | 2.533 | 0.112 | 1.301(0.941-1.798) |
|  |  | Control | 0.076 | 0.783 |  |  |  |  | 3(0.008) | 68(0.172) | 324(0.820) |  |  |  | 74(0.094) | 716(0.906) |  |  |  |
| rs11167764 |  | Case |  |  |  | A | C |  | 4(0.010) | 93(0.242) | 287(0.747) | 2.105 | 0.349 |  | 101(0.132) | 667(0.869) | 1.174 | 0.279 | 0.854(0.642-1.136) |
|  |  | Control | 0.002 | 0.988 |  |  |  |  | 9(0.023) | 101(0.256) | 285(0.722) |  |  |  | 119(0.151) | 671(0.849) |  |  |  |
| rs3212227 |  | Case |  |  |  | G | T |  | 71(0.185) | 188(0.490) | 125(0.326) | 0.420 | 0.811 |  | 330(0.430) | 438(0.570) | 0.145 | 0.704 | 0.962(0.787-1.175) |
|  |  | Control | 0.599 | 0.438 |  |  |  |  | 80(0.203) | 187(0.473) | 128(0.324) |  |  |  | 347(0.439) | 443(0.561) |  |  |  |
| rs6556412 |  | Case |  |  |  | A | G |  | 67(0.175) | 191(0.497) | 126(0.328) | 0.091 | 0.956 |  | 325(0.423) | 443(0.577) | 0.053 | 0.818 | 0.977(0.799-1.194) |
|  |  | Control | 0.010 | 0.919 |  |  |  |  | 72(0.183) | 194(0.492) | 128(0.325) |  |  |  | 338(0.429) | 450(0.571) |  |  |  |
| rs6556416 |  | Case |  |  |  | A | C |  | 7(0.018) | 72(0.188) | 305(0.794) | 2.133 | 0.344 |  | 86(0.112) | 682(0.888) | 1.417 | 0.234 | 1.220(0.879-1.693) |
|  |  | Control | 0.076 | 0.783 |  |  |  |  | 3(0.008) | 68(0.172) | 324(0.820) |  |  |  | 74(0.094) | 716(0.906) |  |  |  |
| rs6871626 |  | Case |  |  |  | A | C |  | 48(0.125) | 149(0.388) | 187(0.487) | 7.395 | 0.025 |  | 245(0.319) | 523(0.681) | 1.177 | 0.278 | 1.127(0.908-1.398) |
|  |  | Control | 2.164 | 0.141 |  |  |  |  | 28(0.071) | 176(0.446) | 191(0.484) |  |  |  | 232(0.294) | 558(0.706) |  |  |  |
| rs6887695 |  | Case |  |  |  | C | G |  | 59(0.154) | 191(0.497) | 134(0.349) | 0.305 | 0.859 |  | 309(0.402) | 459(0.598) | 0.269 | 0.606 | 0.948(0.775-1.161) |
|  |  | Control | 0.188 | 0.665 |  |  |  |  | 66(0.167) | 196(0.496) | 133(0.337) |  |  |  | 328(0.415) | 462(0.585) |  |  |  |
| rs7709212 |  | Case |  |  |  | C | T |  | 76(0.198) | 187(0.487) | 121(0.315) | 0.886 | 0.642 |  | 339(0.441) | 429(0.559) | 0.668 | 0.414 | 0.920(0.754-1.123) |
|  |  | Control | 0.898 | 0.343 |  |  |  |  | 89(0.225) | 187(0.473) | 119(0.301) |  |  |  | 365(0.462) | 425(0.538) |  |  |  |

SNPs: Single nucleotide polymorphisms; HWE: Hardy-Weinberg equilibrium; OR:odds ratio.

*P*-value was survived after Bonferroni correction.


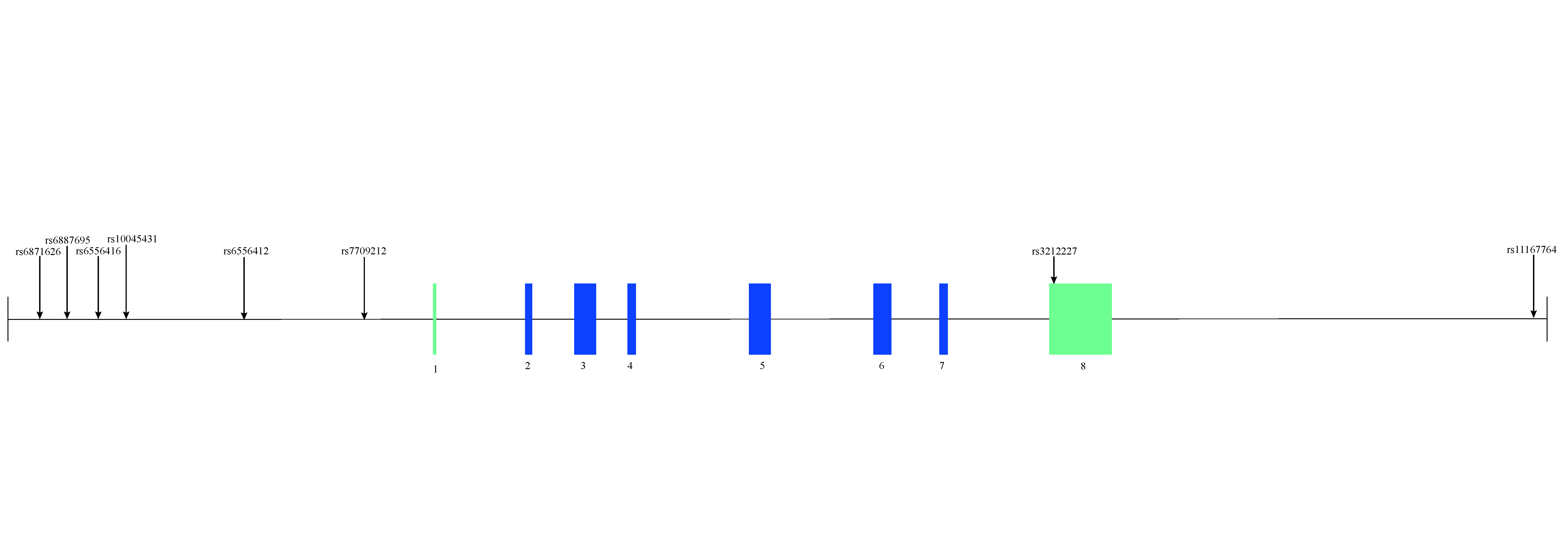


**Figure A. The IL-12B gene structure of the selected SNPs.**


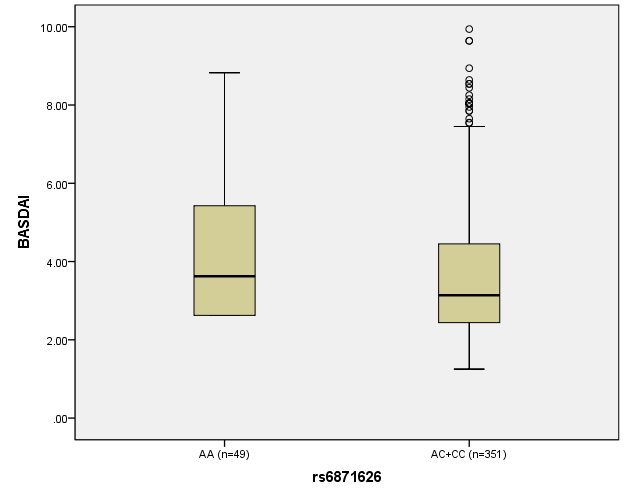


**Figure B. Comparison of the BASDAI scores among different genotypes of rs6871626 in AS patients.**

The AA genotype were significantly correlated with an increased compared to the AC and CC genotypes (*P*-value = 0.045). The mean ± SD for the AA genotype and combined AC and CC genotypes were 4.244 ± 1.713 and 3.655 ± 1.952, respectively.


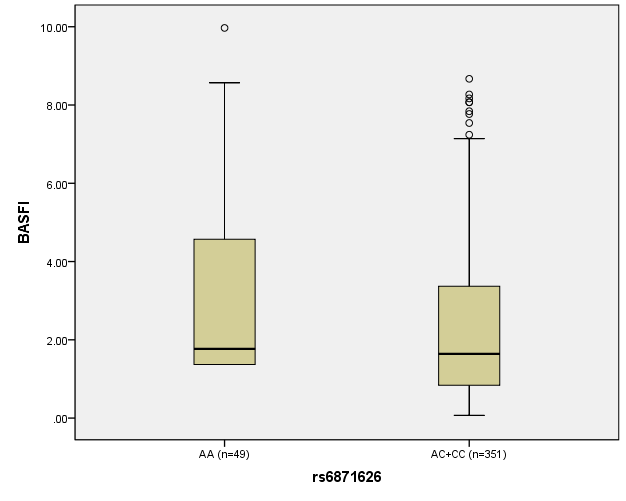


**Figure C. Comparison of the BASFI scores among different genotypes of rs6871626 in AS patients.**

The AA genotype were significantly correlated with an increased compared to the AC and CC genotypes (*P*-value = 0.009). The mean ± SD for the AA genotype and combined AC and CC genotypes were 3.058 ± 2.276 and 2.244 ± 2.012, respectively.


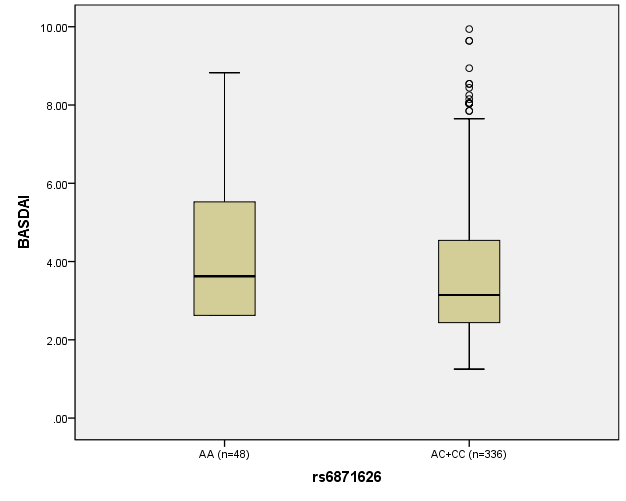


**Figure D. Comparison of the BASDAI scores among different genotypes of rs6871626 in HLA-B27(+) AS patients.**

The AA genotype were significantly correlated with an increased compared to the AC and CC genotypes (*P*-value = 0.046). The mean ± SD for the AA genotype and combined AC and CC genotypes were 4.262 ± 1.726 and 3.668 ± 1.943, respectively.


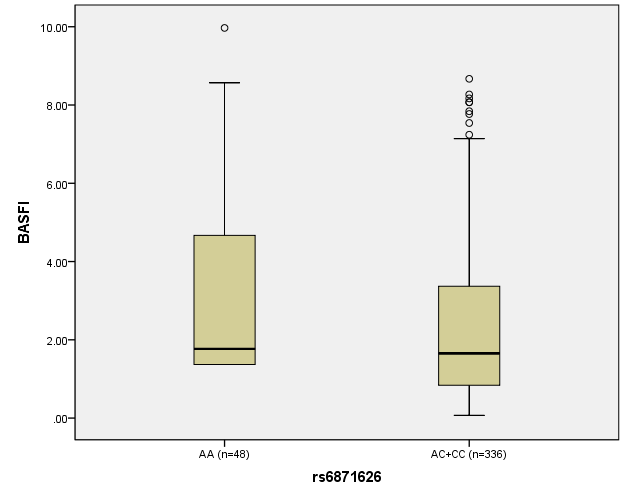


**Figure E. Comparison of the BASFI scores among different genotypes of rs6871626 in HLA-B27(+) AS patients.**

The AA genotype were significantly correlated with an increased compared to the AC and CC genotypes (*P*-value = 0.009). The mean ± SD for the AA genotype and combined AC and CC genotypes were 3.093 ± 2.287 and 2.258 ± 2.034, respectively.
